# An integrated nomogram combining lncRNAs classifier and clinicopathologic factors to predict the recurrence of head and neck squamous cell carcinoma

**DOI:** 10.1038/s41598-019-53811-0

**Published:** 2019-11-25

**Authors:** Jie Cui, Qingquan Wen, Xiaojun Tan, Jinsong Piao, Qiong Zhang, Qian Wang, Lizhen He, Yan Wang, Zhen Chen, Genglong Liu

**Affiliations:** 1 Department of Pathology, Affiliated Cancer Hospital & Institute of Guangzhou Medical University, Guangzhou, 510095 Guangdong Province P.R. China; 2 Department of Head and Neck Surgery, Affiliated Cancer Hospital & Institute of Guangzhou Medical University, Guangzhou, 510095 Guangdong Province P.R. China; 3 Department of Intensive Care Unit, Shunde Hospital, Southern Medical University (The First people’s hospital of Shunde), Foshan, 528308 Guangdong Province P.R. China

**Keywords:** Predictive markers, Head and neck cancer, Risk factors

## Abstract

Long non-coding RNAs (lncRNAs) which have little or no protein-coding capacity, due to their potential roles in the cancer disease, caught a particular interest. Our study aims to develop an lncRNAs-based classifier and a nomogram incorporating the lncRNAs classifier and clinicopathologic factors to help to improve the accuracy of recurrence prediction for head and neck squamous cell carcinoma (HNSCC) patients. The HNSCC lncRNAs profiling data and the corresponding clinicopathologic information were downloaded from TANRIC database and cBioPortal. Using univariable Cox regression and Least absolute shrinkage and selection operator (LASSO) analysis, we developed 15-lncRNAs-based classifier related to recurrence. On the basis of multivariable Cox regression analysis results, a nomogram integrating the genomic and clinicopathologic predictors was built. The predictive accuracy and discriminative ability of the inclusive nomogram were confirmed by calibration curve and a concordance index (C-index), and compared with TNM stage system by C-index, receiver operating characteristic (ROC) analysis. Decision curve analysis (DCA) was conducted to evaluate clinical value of our nomogram. Consequently, fifteen recurrence-free survival (RFS) -related lncRNAs were identified, and the classifier consisting of the established 15 lncRNAs could effectively divide patients into high-risk and low-risk subgroup. The prediction ability of the 15-lncRNAs-based classifier for predicting 3- year and 5-year RFS were 0.833 and 0.771. Independent factors derived from multivariable analysis to predict recurrence were number of positive LNs, margin status, mutation count and lncRNAs classifier, which were all embedded into the nomogram. The calibration curve for the recurrence probability showed that the predictions based on the nomogram were in good coincide with practical observations. The C-index of the nomogram was 0.76 (0.72–0.79), and the area under curve (AUC) of nomogram in predicting RFS was 0.809, which were significantly higher than traditional TNM stage and 15-lncRNAs-based classifier. Decision curve analysis further demonstrated that our nomogram had larger net benefit than TNM stage and 15-lncRNAs-based classifier. The results were confirmed externally. In summary, a visually inclusive nomogram for patients with HNSCC, comprising genomic and clinicopathologic variables, generates more accurate prediction of the recurrence probability when compared TNM stage alone, but more additional data remains needed before being used in clinical practice.

## Introduction

As an aggressive malignancy, head and neck squamous cell carcinoma (HNSCC) arise in the squamous epithelium along the head and neck region, including the nasal cavity, oral cavity and tongue, pharynx (nasal pharynx, oropharynx, hypopharynx) and larynx. In 2018, it is estimated to affect approximately 650 000 people, leading to over 350 000 deaths worldwide annually^1^. It has been reported that the 5-year overall survival rate is approximately 50% for treated HNSCC patients^2^. The current gold-standard therapy protocol consists of radical surgical resection followed by adjuvant radiotherapy as monotherapy, definitive chemoradiotherapy followed by chemotherapy or targeted therapy^3^. Despite advances in the treatment of HNSCC, after curative treatment patients who will develop recurrent can be as high as 50%, which render the major obstacles to long-term survival in HNSCC^4^.

HNSCC is a heterogeneous group, comprising different subsets with distinct outcomes. This heterogeneity may be ascribed to differences in the tumors’ biologic behaviors. Traditional prognostic factors are not helpful in predicting which patients with HNSCC will develop recurrence. Molecular investigation of HNSCC could provide information for predicting recurrence and for triaging the patients who may require and benefit from adjuvant therapies. Hence, identifying reliable and accurate predictive markers/models to screen out which subset of patients with HNSCC is vulnerable to develop recurrence is urgently needed.

As revealed by the previous genomic studies, more than 98% of the human genome is actively transcribed as non-coding RNAs (ncRNAs)^5^. Conventionally, these ncRNA family is roughly classified into two groups based on molecular size: small ncRNA (eg microRNA; the length is <200 nt) and long non-coding RNA (lncRNA; the length is more than 200 nt)^6^. Accumulating evidence has revealed that lncRNAs act as key regulators by participating in gene regulation at the transcriptional, posttranscriptional and chromosomal levels^6^ and are involved in large range of biological processes, particularly in cancers^7,8^. Compared with protein-coding RNAs, the expression patterns of the lncRNAs are more specific, which representing a vast source of largely unstudied potential molecular drivers of human cancer and can be as a new class of novel cancer biomarkers^9^. Previous genomewide studies have investigated the lncRNAs classifier, with accurate prediction value, as a predictor for overall survival (OS)^10–13^, but not for recurrence-free survival (RFS). Because OS is more likely to be influenced by post recurrence treatment and comorbidity, RFS reflects the biologic behavior more precisely for patients with HNSCC. Thus, it will be more practical and valuable to identify specific lncRNAs involved in HNSCC recurrence.

In the current study, we hypothesized that integrated nomogram incorporating genomic and clinicopathologic factors might accurately predict the recurrence of HNSCC. We selected candidate lncRNAs that significantly linked with recurrence outcome and then built a multiple-lncRNAs classifier in the training set. The lncRNAs classifier was further combined with clinicopathological factors to develop an integrated nomogram for predicting recurrence of HNSCC. We assessed the predictive ability and clinical application of the nomogram and compared it to the TNM stage. Additionally, we will validated it in an internal and external validation set.

## Materials and Methods

### Collection of lncRNAs data and clinicopathologic characteristics of HNSCC patients

The lncRNAs profiling data of 502 HNSCC patients and 44 normal controls were downloaded from The Atlas of ncRNA in Cancer (TANRIC)(TCGA) (http://ibl.mdanderson.org/tanric/_design/basic/query.html). The matched clinical parameters, including age, sex, primary site, smoking history, alcohol history, history of other malignancy, history of neoadjuvant treatment, lymph node neck dissection, number of lymph nodes (LNs), number of positive LNs, margin status, tumor grade, clinical T stage, clinical N stage, clinical TNM stage, fraction genome altered, mutation count, and RFS time were obtained from cBioPortal (http://www.cbioportal.org/). The RFS was time from final surgical excision to recurrence. Patients not having a recurrence or those patients who died without recurrence were censored at the time of last follow-up. After removing patients without available RFS information or the unavailability of lncRNAs data, a total of 371 HNSCC patients were used for further analysis. The TNM stage of HNSCC adopted American Joint Committee on Cancer (AJCC) tumor-node -metastasis (TNM) stage system seventh edition on the basis of database provided. HPV status determined by RNA-Seq analysis was consistent with HPV status defined by *in situ* hybridization.p16 staining is an indirect method of HPV detection by immunohistochemical technique, and is considered less accurate than measurement of HPV RNA expression, therefore RNA-Seq analysis was used as a primary measure of HPV status in our analysis. Subsequently, 371 HNSCC patients were randomly assigned to a training set (N = 187) and a validation set (N = 184) by R software. Moreover, GSE65858 dataset (270 HNSCC tissue samples and 30 adjacent non-tumor tissue samples, and 270 tumor samples had complete information of recurrence status and recurrence-free survival time information) from Gene Expression Omnibus (GEO) (https://www.ncbi.nlm.nih.gov/geo/) was used for external validation.

### Construction and validation of lncRNAs classifier for RFS

**Figure 1:**
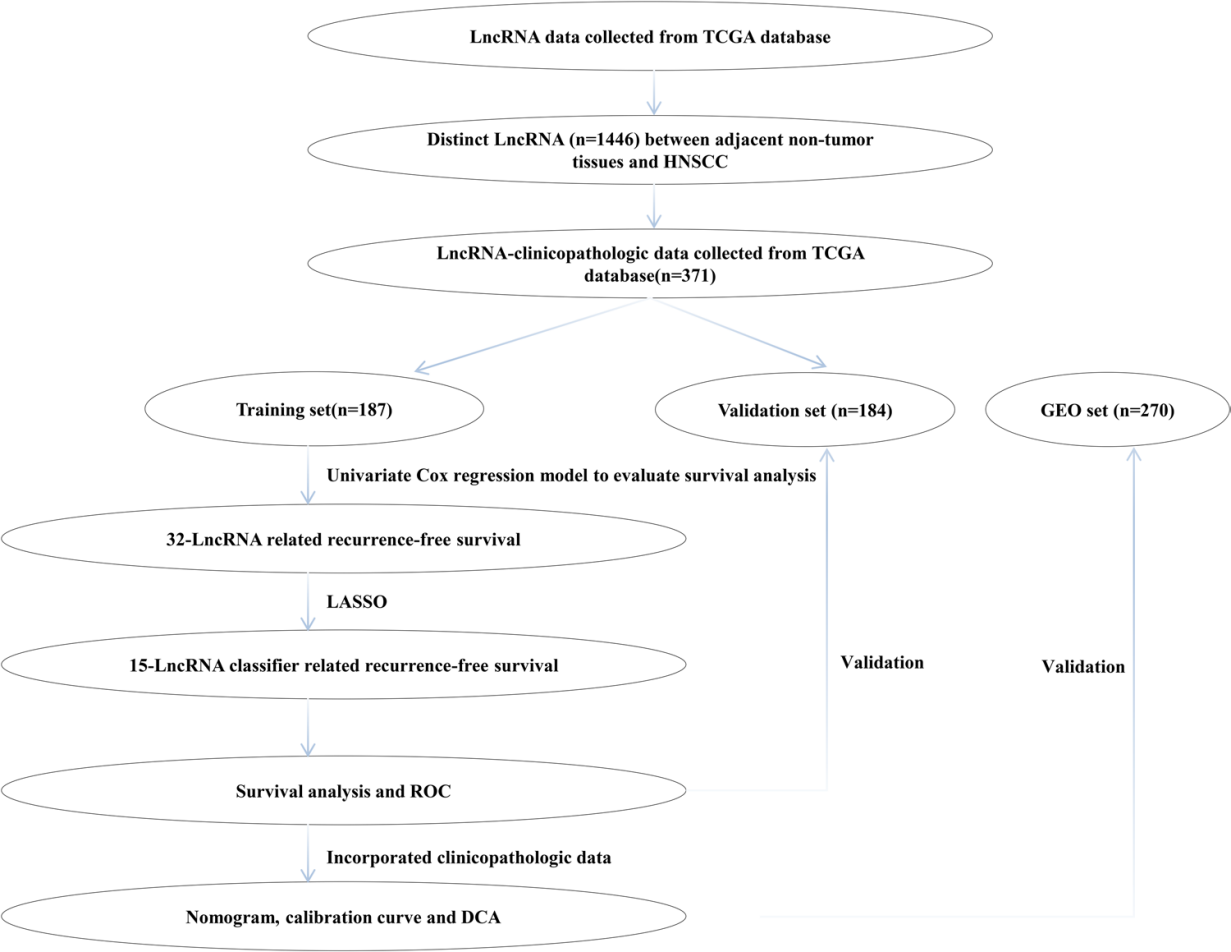
The flowchart of study design. LASSO: least absolute shrinkage and selection operator.

Initially, moderated t-statistics method and Benjamini–Hochberg procedure were used to identify distinct lncRNAs between HNSCC tissues and normal tissues. The cut-off criteria of differential lncRNAs was ***P*** < 0.05 and the false discovery rate (FDR) < 0.05. Then univariate Cox regression analysis was used to select RFS-related lncRNAs in the training set (***P*** < 0.05). After primary filtration, Least absolute shrinkage and selection operator (LASSO) logistic regression analysis^14^, with penalty parameter tuning conducted by 10-fold cross-validation, was built to pick out candidate lncRNAs, and final performed L1 penalized Cox analysis to further narrow lncRNAs in the training set^15^. After layers of screening, these eligible lncRNAs was constructed a classifier. According to the expression levels of each sample and corresponding coefficients for each of them, we calculated the risk scores of HNSCC patients and then divided patients into high-risk and low-risk subgroup based on the optimal cut-of value, which was chosen with the maximal sensitivity and specificity in receiver operating characteristic (ROC) curve (time-independent) in the training set. The RSF difference between high-risk group and low-risk group were further compared by the Kaplan-Meier analysis. Meanwhile, ***P***-values and hazard ratio (HR) with 95% confidence interval (CI) were generated by Log-rank tests. Additionally, considering the human papillomavirus (HPV) is very important parameter for HNSCC patients, we performed a sensitivity analysis by excluding these cases of oropharynx. Furthermore, stratified analysis base on various clinical characteristics (eg. HPV status, TNM stage) is conducted to evaluate the discrimination ability of lncRNAs signature in TCGA cohort and in GEO cohort, respectively. Given HPV variables existing missing value in TCGA cohort, we perform stratified analysis in entire dataset.The flowchart of the present study was shown in Fig. 1.

### Development and validation of genomic-clinicopathologic nomogram

To build a genomic-clinicopathologic nomogram, we used univariate and multivariate Cox regression analysis to identify clinical risk parameters associated with RFS in the training set. Then, the lncRNAs classifier, together with the risk parameters, were used to develop an integrated nomogram in the training set.

The performance of model was evaluated by the calibration and discrimination. Discrimination is the models ability to distinguish between patients who recur from HNSCC and patients who will not. The concordance index (C-index) was calculated to evaluate the discrimination. Besides, based on the score generated by the nomogram, we illustrated discrimination by dividing the dataset into three groups. We plotted a Kaplan–Meier curve for all three groups. In additional, calibration curves were assessed graphically by plotting the observed rates against the nomogram predicted probabilities.

ROC analysis was used to assess and compare the discrimination ability of the nomogram with TNM stage and lncRNAs-based classifier. Clinical usefulness and net benefit of the predictive models were estimated with decision curve analysis (DCA)^16^ and compared to traditional TNM stage or lncRNAs classifier.

### Sample size

To develop a prediction nomogram with time-to-event data, the sample size should be based on the events-per-variable (EPV). This must be greater than or equal to 10. In our sample there were a total of 77 recurrences, which allows us to construct a prediction nomogram with a maximum of six predictors (EPV = 62/6 = 10.3 ≥ 10) in the training cohort and a maximum of seven predictors (EPV = 77/7 = 11 ≥ 10) in validation cohort,

### Statistical analysis

Normally distributed data were described as mean (standard deviation [SD]) whereas non-normally distributed data were expressed as median (interquartile ranges [IQR]). Categorical variables are provided as proportions (%).After classifying the patients with cancer recurrence, we calculated the best cutoff values of number of Lymph nodes, number of positive LNs, mutation count and fraction genome altered, which was a point when the Youden index (sensitivity + specificity − 1) reached the maximum value through receiver operating curve (ROC) analysis.

If there were missed values in some of the potential predictors, these missing data would be imputed, as full case analysis would improve the statistical power and reduce potentially biassed result^17^. Multiple imputation was used to interpolate the missing data as the missing data were considered missing at random after analyzing patterns of them^18^.

LASSO analysis was performed with “glmnet” packages, and ROC analysis was done with “timeROC” and “survivalROC” packages. The nomogram and calibration plots were generated with “rms” packages, and DCA was performed with the “stdca.R”.

SPSS statistics 22.0 and R software (R version 3.5.2) were used to conduct the statistical analysis. A two sided ***P*** < 0.05 would be recognized as statistically significant.

### Ethics approval and consent to participate

Institutional ethical approval was not required as data was acquired from publicly available databases TANRIC and cBioPortal, and the Written informed consents had been attained from the patients before our study.

## Results

### Demographic parameters and RFS outcome of HNSCC patients

In the current study, 371 HNSCC patients with available lncRNAs data and corresponding clinicopathologic information were included. The basic clinicopathologic characteristics of HNSCC patients were summarized in Table 1. The median follow-up times of 20.83 months (range: 1.81 to 180.03 months) and 20.17 months (range: 1.51 to 172.54 months) for the training and validation cohorts, respectively. Of all the 371 LSCC patients, 139 patients (37.5%) developed recurrence during follow-up. The estimated 3-year and 5-year RFS rates were 64% (56.2–71.8%) and 55.4% (44.4–66.4%) in the training set, respectively. Similarly, the estimated 3-year and 5-year RFS rates were 57.6% (49.6–65.6%) and 47.3% (37.1–57.5%) in the validation set, respectively.

**Table 1:** Characteristics of patient in the training set and validation set from TANRIC (n = 371).

Variable	Category	Training set		Validation set	
		(n = 187)	%	(n = 184)	%
Age (years)	Median	60		60	
	Range (Years)	19–85		28–88	
	NA	0	0	0	0
Sex	Male	142	75.9	142	77.2
	NA	0	0	0	0
Primary site	Larynx	39	20.9	42	22.8
	Oral tongue	52	27.8	45	24.5
	Oral cavity	20	10.7	18	9.8
	Others	76	40.6	79	42.9
	NA	0	0	0	0
Smoking history	Yes	75	40.1	79	42.9
	NA	1	0.5	2	1.1
Alcohol history	Yes	129	69	130	70.7
	NA	3	1.6	5	2.7
History of other malignancy	Yes	13	7.0	9	4.9
	NA	0	0	0	0
History of neoadjuvant treatment	Yes	2	1.1	0	0
	NA	0	0	0	0
Lymph node neck dissection	Yes	164	87.7	150	81.5
	NA	1	0.5	1	0.5
Number of Lymph nodes	≤45	124	66.3	111	60.3
	>45	49	26.2	46	25
	NA	14	7.5	27	14.7
Number of positive LNs	<3	130	69.5	116	63
	≥3	42	22.5	41	22.3
	NA	15	8.0	27	14.7
Margin status	Negative	135	72.2	143	77.7
	Positive	32	17.1	21	11.4
	NA	20	10.7	20	10.9
Tumor grade	G1-G2	139	74.3	128	69.6
	G3-G4	41	21.9	55	29.9
	NA	7	3.7	1	0.5
Clinical T stage	T1-T2	69	36.9	62	33.7
	T3-T4	114	61	113	61.4
	NA	4	2.1	9	4.9
Clinical N stage	N0	83	44.4	90	48.9
	N1-N3	97	51.9	83	45.1
	NA	7	3.7	11	6.0
Clinical TNM stage	I-II	39	20.9	42	22.8
	III-IV	145	77.5	133	72.3
	NA	3	1.6	9	4.9
Mutation count	≤65	41	21.9	37	20.1
	>65	146	78.1	147	79.9
	NA	0	0	0	0
Fraction genome altered	≤ 0.29	125	66.8	126	68.5
	>0.29	61	32.6	55	29.9
	NA	1	0.5	3	1.6

Abbreviations: NA = not available, LN = lymph nodes.

### Development and validation of lncRNAs-based classifier

**Figure 2:**
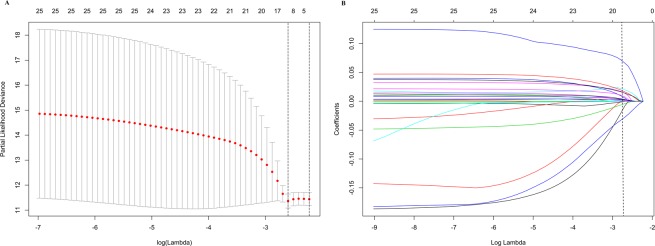
(**A**) fifteen lncRNAs selected by LASSO Cox regression analysis. The two dotted vertical lines are drawn at the optimal values by minimum criteria (left) and 1 - s.e. criteria (right). (**B**) LASSO coefficient profiles of the 26 lncRNAs. A vertical line is drawn at the optimal value by minimum criteria and results in fifteen non-zero coefficients. Fifteen lncRNAs—AC012531.2, AC020551.1, AC020637.1, AC076966.1, AC079789.1, AC090826.2, AC092132.1, AC097521.2, AC104051.2, AC145207.3, ADARB2.AS1, AL122019.1, AL138974.1, ATP6V1B1.AS1, LINC02471—with coefficients −0.02235, 0.01734, 0.00017, −0.00203, 0.06052, −0.00037, 0.00943, 0.00188, 0.01343, 0.00086, 0.00513, 0.00285, 0.01173, 0.00176, 0.00116, respectively, were selected in the LASSO Cox regression model.

First, 1446 distinct lncRNAs between HNSCC tissues and normal tissues were obtained basing on the filter criteria described on the section of Methods **(**Supplementary Material 1**)**. Then, using univariable Cox regression analysis, we identified 32 RFS related lncRNAs in the training set **(**Supplementary Material 2**)**. Next, the selected 32 RFS related lncRNAs were entered into LASSO logistic regression model and 26 had non-zero coefficients (Fig. S1).Final, we used a LASSO Cox regression model to further narrow down RFS-related lncRNAs for patients with HNSCC in the training cohort, which were AC012531.2, AC020551.1, AC020637.1, AC076966.1, AC079789.1, AC090826.2, AC092132.1, AC097521.2, AC104051.2, AC145207.3, ADARB2.AS1, AL122019.1, AL138974.1, ATP6V1B1.AS1, LINC02471 (Fig. 2A,B).On the basis of the coefficients weighted by LASSO Cox regression analysis, a classifier was developed, and the risk score was as follows: risk score = (−0.02235* AC020637.1) + (0.01734* AC020551.1) + (0.00017* AC020637.1) + (−0.00203* AC076966.1) + (0.06052* AC079789.1) + (−0.00037* AC090826.2) + (0.00943* AC092132.1) + (0.00188* AC097521.2) + (0.01343* AC104051.2) + (0.00086 * AC145207.3) + (0.00513* ADARB2.AS1) + (0.00285* AL122019.1) + (0.01173* AL138974.1) + (0.00176* ATP6V1B1.AS1) + (0.00116* LINC02471). Using ROC curve to generate the optimal cutoff value for the risk score, patients were categoried into high-risk group and low-risk group. As was shown at Fig. S2, patients with high risk score were more likely to develop recurrence and had shorter RFS than those with low risk score in the training set (5.93 vs 29.2 months, HR = 4.92, 95%CI: 2.98–8.09, ***P*** < 0.0001)(Fig. S3A). Likewise, the lncRNAs classifier could also classify patients into the high-risk and the low-risk subgroup by the same cut-off value in the internal validation set and the external validation set. The median RFS time of high-risk patients was shorter than low-risk patients in the internal validation set (14.22 vs 27.2 months, HR = 1.941, 95%CI: 1.28–2.94, P < 0.0001) (Fig. S3B), the external validation set (12.12 vs 54.6 months, HR = 6.735, 95%CI: 3.802–11.93, P < 0.0001) (Fig. S3C). Additionally, the lncRNAs classifier showed favorable predictive efficacy, with AUC of 0.833 (3 year RFS) and AUC of 0.771 (5 year RFS) in the training cohort, as well as with AUC of 0.695 (3 year RFS) and AUC of 0.718 (5 year RFS) in the internal validation cohorts, as well as with AUC of 0.846 (3 year RFS) and AUC of 0.79 (5 year RFS) in the external validation cohort, respectively (Fig. S3D–F). Furthermore, we performed a sensitivity analysis by excluding these cases of oropharynx. As Fig. S4 show, the LncRNAs classifier showed similar predictive efficacy between non-oropharynx HNSCC patients and entire HNSCC patients, with AUC of 0.822 (3 year RFS) and AUC of 0.756 (5 year RFS) in the training cohort as well as with AUC of 0.717 (3 year RFS) and AUC of 0.701 (5 year RFS) in the internal validation cohorts. Finally, 15 lncRNAs signature in subsets of patients with different clinical variables were analyzed by stratification analysis in TCGA cohort and GEO cohort. When stratified according to clinical variables (HPV status, TNM stage), 15 lncRNAs signature remained a clinically and statistically significant prognostic model in TCGA cohort (P < 0.0001) (Fig. S5) and in GEO cohort (P < 0.0001) (Fig. S6).

### Development and Validation of genomic-clinicopathologic nomogram

**Figure 3:**
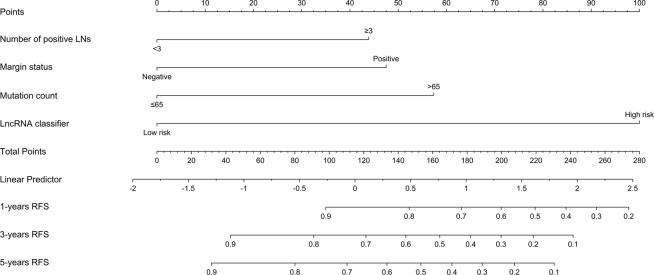
(**A**) Nomogram for predicting1-year, 3-year and 5-year RFS probability of HNSCC after radical surgery. To estimate risk, calculate points for each variable by drawing a straight line from patient’s variable value to the axis labeled “Points.” Sum all points and draw a straight line from the total point axis to the1-year, 3-year and 5-year RFS axis.

**Figure 4:**
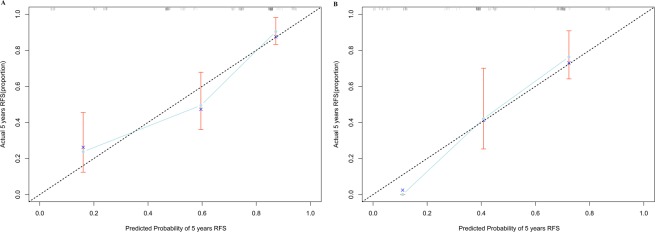
(**A**,**B**) ROC curves compare the prognostic accuracy of the nomogram with TNM staging or lncRNAs classifier in predicting survival probability in the training set and in the validation set. (**C**,**D**) Decision curve analysis for the nomogram, TNM staging and lncRNAs classifier in prediction of recurrence of patients in the training set and in the validation set.

Using univariate Cox analysis, we identified four variables, including number of positive LNs, margin status, mutation count and lncRNAs classifier, were associated with RFS in the training set (Table 2). Multivariable analysis continued to verify that number of positive LNs, margin status, mutation count and lncRNAs classifier, were independent risk factors for RFS in the training set. On the basis of the multivariate analysis of RFS, we built genomic-clinicopathologic nomogram to predict1-year, 3-year and 5-year RFS (Fig. 3). The C-index of the integrated nomogram was 0.76 (0.72–0.79) (Table 3) and the calibration plots exhibited good consistency between the predicted probability and the actual probability for 3-year and 5-year RFS (Figs. 4A and S7A).Likewise, consistent results were also found in the validation set. The C-index of the integrated nomogram in the validation set was 0.74 (0.71–0.76) (Table 3), and also showed good coincide between the predicted RFS and the actual RFS (Figs. 4B and S7B). Besides, the tertiles of all the total points were used to divide the patients into high-, intermediate- and low-risk groups. The Kaplan-Meier analysis (Log-rank *P* < 0.0001) of the three risk subgroups indicated the great utility of the integrated nomogram in training set (Fig. S8A) and in validation set (Fig. S8B).

**Table 2:** Univariable and multivariable Cox regression analysis for prediction of RFS.

Factors	Subgroup	Univariable analysis		Multivariable analysis	
		HR (95%CI)	*P*	HR (95%CI)	*P*
Age		1.02 (0.99–1.04)	0.139	NA	NA
Sex	Female	1			
	Male	0.76 (0.44–1.31)	0.324	NA	NA
Primary site	Larynx	1			
	Oral tongue	1.20 (0.54–2.66)	0.663	NA	NA
	Oral cavity	1.43 (0.53–3.77)	0.469	NA	NA
	Others	1.77 (0.86–3.62)	0.121	NA	NA
Smoking history	No	1			
	Yes	0.76 (0.45–1.27)	0.295	NA	NA
Alcohol history	No	1			
	Yes	1.66 (0.90–3.07)	0.107	NA	NA
History of other malignancy	No	1			
	Yes	1.05 (0.42–2.62)	0.917	NA	NA
Lymph node neck dissection	Yes	1			
	No	1.08 (0.46–2.50)	0.865	NA	NA
Number of Lymph nodes	≤45	1			
	>45	1.35 (0.78–2.31)	0.284	NA	NA
Number of positive LNs	<3	1		1	
	≥3	2.95 (1.79–4.88)	0.000*	1.90 (1.12–3.21)	0.017*
Margin status	Negative	1		1	
	Positive	2.87 (1.71–4.81)	0.000*	2.11 (1.24–3.61)	0.06*
Tumor grade	G1-G2	1			
	G3-G4	0.86 (0.47–1.58)	0.63	NA	NA
Clinical T stage	T1-T2	1			
	T3-T4	1.48 (0.85–2.58)	0.162	NA	NA
Clinical N stage	N0	1			
	N1-N3	1.25 (0.75–2.09)	0.40	NA	NA
Clinical TNM stage	I-II	1			
	III-IV	1.64 (0.81–3.34)	0.170	NA	NA
Mutation count	≤65	1			
	>65	2.34 (1.06–5.13)	0.035*	2.56 (1.14–5.72)	0.022*
Fraction Genome altered	≤0.29	1			
	>0.29	1.10 (0.65–1.86)	0.724	NA	NA
LncRNA classifier	Low risk	1		1	
	High risk	4.97 (2.64–9.35)	0.000*	4.72 (2.48–9.0)	0.000*

Abbreviations: HR = hazard ratio, CI = confidence intervals.

NOTE: NA, not available. These variables were eliminated in the multivariate Cox regression model, so the HR and *P* values were not available. ^*^P < 0.05.

**Table 3:** Assessing the prediction performance of the TNM stage, LncRNA classifier and nomogram in training set and validation set.

Cohort	Model	Homogeneity monotonicity and discriminatory ability			Akaike information criterion (AIC)****
		Likelihood ratio (LR) test^*^	Linear trend χ2 test**	C-index (95% CI)^***^	
Training set	TNM stage	4.5	4.3	0.57 (0.52–0.59)	593
	LncRNA classifier	32	30.5	0.67 (0.64–0.70)	561
	Nomogram	58.1	62.7	0.76 (0.72–0.79)	541
Validation set	TNM stage	5.9	4.8	0.55 (0.52–0.58)	711
	LncRNA classifier	20.4	20.4	0.63 (0.61–0.65)	689
	Nomogram	58.1	69.5	0.74 (0.71–0.76)	661

Assessing the prognostic performance of the TNM stage, lncRNAs classifier and nomogram.

^*^Higher homogeneity likelihood ratio indicates a smaller difference within the staging system, it means better homogeneity.

^**^Higher discriminatory ability linear trend indicates a higher linear trend between staging system, it means better discriminatory ability and gradient monotonicity.

^***^A higher c-index means better discriminatory ability.

^****^Smaller AIC values indicate better optimistic prognostic stratification.

### Comparison of predictive performance and clinical usefulness between nomogram and TNM stage or lncRNAs classifier

**Figure 5:**
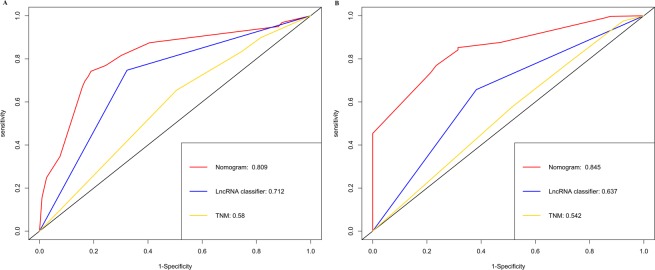
ROC curves compare the prognostic accuracy of the nomogram with TNM staging or lncRNAs classifier in predicting survival probability (**A**) in the training set and (**B**) in the validation set.

To further evaluate the predictive ability of the genomic-clinicopathologic nomogram, we compared the C-index and ROC analysis results of integrated nomogram with TNM stage and lncRNAs classifier in the training set and validation set. As was shown at Table 3, the C-index of integrated nomogram was higher than that of TNM stage (0.57 (0.52–0.59) in the training set, and 0.55 (0.52–0.58) in the validation set) and the lncRNAs classifier (0.67 (0.64–0.70) in the training set, and 0.63 (0.61–0.65) in the validation set). Likelihood ratio test, linear trend χ2 test and akaike information criterion all demonstrated that the integrated nomogram had better prediction efficiency than the TNM stage or lncRNAs classifier alone. Similar to C-index, ROC analysis also indicated that the integrated nomogram (AUC 0.809 for the training set, and 0.845 for the validation set) was better than TNM stage (AUC 0.58 for the training set, and 0.542 for the validation set) or lncRNAs classifier (AUC 0.712 for the training set, and 0.637 for the validation set) alone in predicting RFS (Fig. 5A,B).

**Figure 6:**
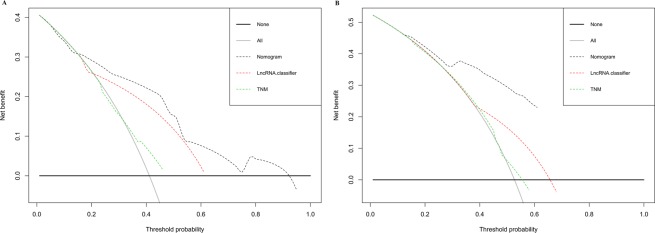
Decision curve analysis for the nomogram, TNM staging and lncRNAs classifier in prediction of recurrence of patients (**A**) in the training set and (**B**) in the validation set.

Finally, DCA was used to compare the clinical usability of the integrated nomogram to that of traditional TNM stage and lncRNAs classifier. Based on a continuum of potential thresholds for death (x axis) and the net benefit of using the model to risk-stratify patients (y axis) relative to assuming all patients will recur, the DCA graphically presented that the nomogram was better than traditional TNM stage or lncRNAs classifier (Fig. 6A,B).

## Discussion

Analyzing HNSCC lncRNAs profiling data and corresponding clinicopathologic variables of 371 HNSCC patients from TANRIC and cBioPortal, we identified fifteen lncRNAs relevant to RFS. According to these lncRNAs, we developed a lncRNAs classifier, which could accurately classified patients into high-risk group and low-risk group. Additionally, we developed a visually integrated nomogram, combining lncRNAs classifier and clinicopathologic parameter to predict recurrence in HNSCC patients underwent surgery resection. The nomogram effectively predicted recurrence risk, with a bootstrapped corrected C-index of 0.76 and AUC of 0.809, which presented better predictive ability and clinical usability than TNM stage alone.

A vast of studies have found that lncRNAs may be exploited as potential effective biomarkers in diagnosis, progression and prognosis of HNSCC^19–23^.

Analyzing Sixty-five HNSCC formalin-fixed and paraffin-embedded samples, Guan *et al*.^19^ revealed that H19 was significantly overexpressed in HNSCC cancer cells and patients in in contrast to adjacent normal specimens. Higher expression of H19 was correlated with tumor recurrence and is considered as prognostic factors for disease free survival, regardless of other confounders. A study in 19 HNSCC patients by Haque *et al*.^20^, using a quantitative real-time polymerase chain reaction array that interrogates lncRNA with established involvement in numerous cancers, uncovered that low MEG3 expression of seven differential expression lncRNA, including SPRY4-IT1, HEIH, LUCAT1, LINC00152, HAND2-AS1, MEG3, and TERC, was related to more favorable 3-year RFS. A study of lncRNAs microarray by Wu *et al*.^21^ found that high expression of lncRNA LOC541471 was significantly related with risk of perineural invasion and lymph node metastasis classification. According to multivariate Cox regression analysis, high expression of lncRNA LOC541471 was an independent predictor for poor RFS. Recently, Diao *et al*.^22^ identified ZEB2-AS1 as a putative oncogenic lncRNA and a novel prognostic biomarker in HNSCC, revealed that overexpression of ZEB2-AS1 associates with tumor aggressiveness and unfavorable prognosis. Notably, Troiano *et al*.^23^ performed a meta-analysis systematically and quantitatively to evaluate prognostic value of lncRNA HOTAIR in HNSCC, verified that high expression of HOTAIR, as a biomarker of aggressiveness, was linked with lymph-node metastasis (odds ratio (OR), 3.31; 95% CI: [1.24, 8.79]; ***P*** = 0, 02). These studies hinted the potential clinical implications of lncRNA in improving the recurrence prediction of HNSCC. Nevertheless, small numbers of patients and single lncRNA with an unacceptable level of suitability or precision limited the clinical applications. A classifier, comprising multiple lncRNAs, can remarkably enhance the accuracy of prediction in various cancers, such as breast cancer, hepatocellular carcinoma and gastric cancer^24–26^. It should be noted that the lncRNAs classifier predicting the RFS outcome of HNSCC has not been reported yet.

To the best of our knowledge, this is the first study constructed an inclusive nomogram, combining lncRNAs classifier and clinicopathologic factors, for predicting recurrence probability in patients with HNSCC. We built a lncRNAs classifier, consist of AC012531.2, AC020551.1, AC020637.1, AC076966.1, AC079789.1, AC090826.2, AC092132.1, AC097521.2, AC104051.2, AC145207.3, ADARB2.AS1, AL122019.1, AL138974.1, ATP6V1B1.AS1, and LINC02471, could effectively categorized patients into high-risk status with shorter RFS and low-risk status with longer RFS. In additional, we identified four independent predictors, namely, number of positive LNs, margin status, mutation count and lncRNAs classifier, which were all assembled into the nomogram. In this study, in consideration of homogeneity, and ability of discrimination and risk stratification of the model, the performance of the nomogram in predicting recurrence ability is superior to the TNM staging system. The strength of the current nomogram is that it integrated genomic and clinicopathological variables, which are important for predicting recurrence risk, but cannot be adopted by TNM stage system. Remarkably, DCA results showed that HNSCC recurrence-related treatment decision based on the nomogram led to more net benefit than treatment decision based on TNM stage, or treating either all patients or none. Taken together, the present nomogram would be clinically useful for the clinicians in tailoring recurrence-associated treatment decision.

Among the fifteen RFS-related lncRNA, ADARB2.AS1, and LINC02471 have been previously reported to be related with cancers, including breast cancer, pancreatic ductal adenocarcinoma and papillary thyroid carcinoma^27–29^. ADARB2-AS1, with highest k-core score, was recognized as core genes in HER-2-enriched subtype breast cancer, which might hopefully become novel molecular biomarkers and therapeutic targets^27^. Subsequently, Permuth *et al*.^28^, analyzing plasma from 57 intraductal papillary mucinous neoplasms (IPMNs) IPMN cases and 24 non-diseased controls frequency-matched by age-group and gender, appraised an 8-lncRNA signature (ADARB2-AS1, ANRIL, GLIS3-AS1, LINC00472, MEG3, PANDA, PVT1, and UCA1) which possessed greater accuracy than standard clinical and radiologic features in differentiating indolent/benign IPMNs from aggressive/malignant IPMNs than standard clinical and radiologic features. Cai *et al*.^29^, using the Cancer Genome Altas (TCGA) database, uncovered that LINC02471 was closely associated with the tumor stage, lymph node metastasis, metastasis and pathological stage of papillary thyroid carcinoma, which could reflect behavior of tumor progression in a more exact way and could function as molecule biomarkers for tumor progression and prognosis. However, other LncRNA (AC012531.2, AC020551.1, AC020637.1, AC076966.1, AC079789.1, AC090826.2, AC092132.1, AC097521.2, AC104051.2, AC145207.3, AL122019.1, AL138974.1, and ATP6V1B1.AS1), which maybe provide new insights into HNSCC development and progression, have not been thoroughly investigated. Hence, further characterization of molecules should be detected to explore potential application value.

Consistent with previous trials, number of positive LNs, was associated with higher risk of recurrence among patients with postoperative HNSCC, which is in agreement with other studies^30,31^. According to ROC analysis, we selected 3 as optimum cut-off point, more than 3 positive LNs is an independent risk factors for recurrence. Recently, Zumsteg *et al*.^32^ found that there was no benefit from postoperative adjuvant chemoradiation in patients with 0–2 positive LNs, while more than 3 positive LNs can significantly benefit from postoperative adjuvant chemoradiation. What’s more, the author discovered association between number of lymph node burden and the efficacy of postoperative adjuvant chemoradiation have an approximate positive linear trend. Similarly, margin status and mutation count were frequently reported risk factors of recurrence for patients with HNSCC, including oral cavity, oropharyngeal cancers, laryngeal carcinoma and so on^33–35^. In addition to these clinicopathologic factors, as expected, the lncRNAs classifier was an effective independent risk variables for the recurrence of patients with HNSCC.

Although our nomogram demonstrated impressive performance in LSCC recurrence prediction, there are specific limitations associated with our trial. First, the presented nomogram based only on single public database, are not yet suitable for general use prior to validation of the predictive models with external datasets. So external and multicenter prospective cohorts with large sample sizes are still needed to validate the clinical application of our model.

Second, Missing variables were a source of defect in this evaluation. We did not investigate identified factors associated with recurrence, such as extracapsular spread^30,34^, lymphovascular invasion status^34^, perineural invasionas^34^ and human papillomavirus (HPV)^36^ as important parameters for HNSCC patients, weren’t well recorded in database. A recent large study, using centralized testing and controlling for other risk factors, examined the prognostic utility of HPV biomarkers among HNSCC across different global regions^37^. HPV positivity were strong biomarkers for improved survival among HNSCC. In additional, HPV positive patients were sensitive to radiotherapy and chemotherapy as well as showed superior survival^38,39^. Hence, we recommend that future studies should added value of those factors in a multivariable prediction model to further improve the accuracy of prediction in HNSCC patients.

Third, our study included a variety of tumors in the head and neck region, such as oral cavity, tongue oropharynx, oral tongue, hypopharynx, larynx cancer and so on. Though they stemmed from epithelial squamous cells, there existing marked heterogeneity between them. On account of lack of enough simple size for a specific tumor, with less more 100 patients for single cancer, we cannot constructed specific nomogram to estimate conditional risk of type-specific recurrence, which maybe reduce the accuracy of prediction. Even so, our estimation based on the predictive nomogram yielded similar C-index on the validation datasets and was significantly superior to TNM stage for recurrence prediction.

Fourth, we do not explore the underlying biological function and pathways of the lncRNAs, so further studies are needed to uncover the related mechanisms.

## Conclusion

We have built visually comprehensive nomogram, incorporated genomic and clinicopathologic factors, for the prediction of recurrence in patients with HNSCC. It seem to be a more effective tool for HNSCC recurrence prediction, compared to TNM stage in terms of the predictive value and clinical usability. The integrated nomogram may help clinicians to make more fitly individualized therapeutic strategies for HNSCC patients.

## Supplementary information

Supplementary Info

## Data Availability

The data that support the findings of this study are provided in Supplementary Materials and is also made available in the TANRIC (http://ibl.mdanderson.org/tanric/_design/basic/query.html), cBioPortal (http://www.cbioportal.org/) and GEO (https://www.ncbi.nlm.nih.gov/geo/).
