# Supplementary material for: An integrated nomogram combining lncRNAs classifier and clinicopathologic factors to predict the recurrence of head and neck squamous cell carcinoma
Source: Sci Rep. 2019 Nov 25;9:17460. doi: 10.1038/s41598-019-53811-0 (PMC6877726; doi:10.1038/s41598-019-53811-0)

An integrated nomogram combining lncRNAs classifier and clinicopathologic factors to predict the recurrence of head and neck squamous cell carcinoma

Jie Cui[2*], Qingquan Wen[2*], Xiaojun Tan[1], Jinsong Piao[1], Qiong Zhang[1], Qian Wang[1], Lizhen He[1], Yan Wang[1], Zhen Chen[3], Genglong Liu[1]

[1] Department of Pathology, Affiliated Cancer Hospital & Institute of Guangzhou Medical University, Guangzhou, 510095, Guangdong Province, PR China.

[2] Department of Head and Neck Surgery, Affiliated Cancer Hospital & Institute of Guangzhou Medical University, Guangzhou, 510095, Guangdong Province, PR China.

[3] Department of Intensive Care Unit, Shunde Hospital, Southern Medical University (The First people's hospital of Shunde), Foshan, 528308, Guangdong Province, PR China.

*These authors contributed equally to this work.

Corresponding author

Genglong Liu, Department of Pathology, Affiliated Cancer Hospital & Institute of Guangzhou Medical University, Guangzhou, 510095, Guangdong Province, PR China, Email: lglong3@mail2.sysu.edu.cn

Jie Cui, Email: cuijir8905@163.com

Qingquan Wen, Email: winquan2003@aliyun.com

Xiaojun Tan, Email: 13022034283@163.com

Jinsong Piao, Email: 9140328@qq.com

Qiong Zhang, Email: 569959211@qq.com

Qian Wang, Email: 13798040957@163.com

Lizhen He, Email: 64938794@qq.com

Yan Wang, Email: wyy7010@sina.com

Zhen Chen, Email: jeanyz@foxmail.com

FigureS1: 26 lncRNAs selected by LASSO logistic regression analysis. A: Selection of tuning parameter (λ) by ten-time cross-validation in LASSO logistic regression model. B: LASSO coefficient profiles of the 26 RSF-associated lncRNAs.

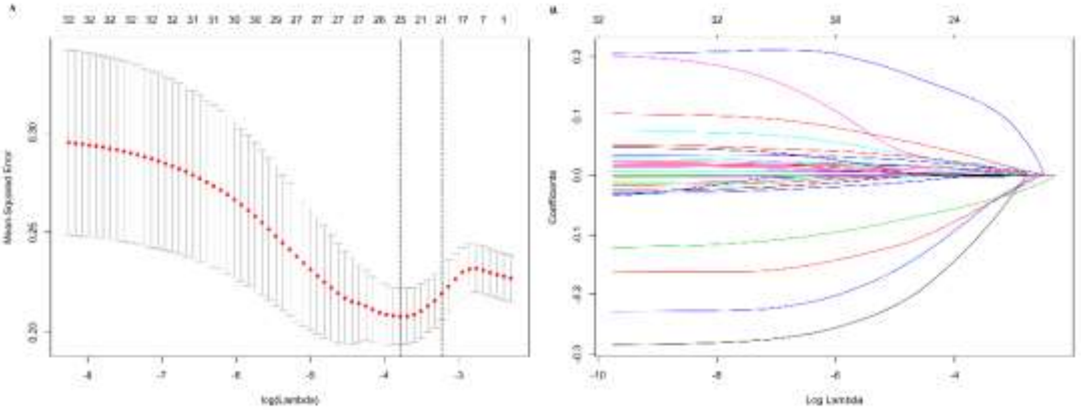

FigureS2: Development and validation of lncRNAs classifier for prediction of recurrence in HNSCC patients. A and D: Distribution of lncRNAs-based classifier risk score in the training set. B and E: Distribution of lncRNAs-based classifier risk score in the internal validation set. C and F: Distribution of lncRNAs-based classifier risk score in the external validation set.

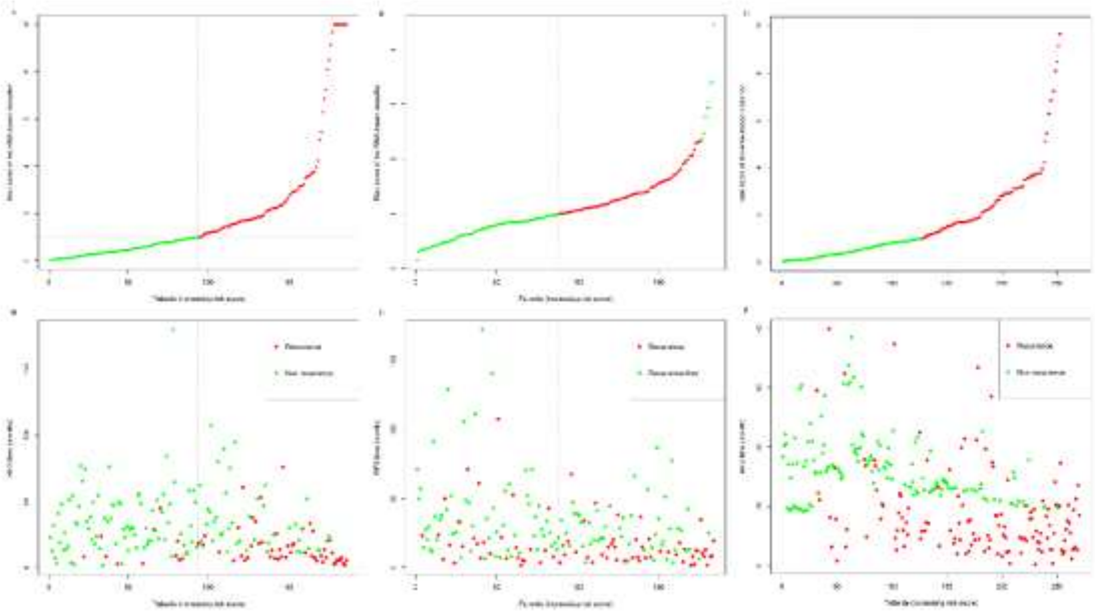

FigureS3: Development and validation of lncRNAs classifier for prediction of recurrence in HNSCC patients. A and D: Distribution of lncRNAs-based classifier risk score in the training set. B and E: Distribution of lncRNAs-based classifier risk score in the internal validation set. C and F: Distribution of lncRNAs-based classifier risk score in the external validation set.

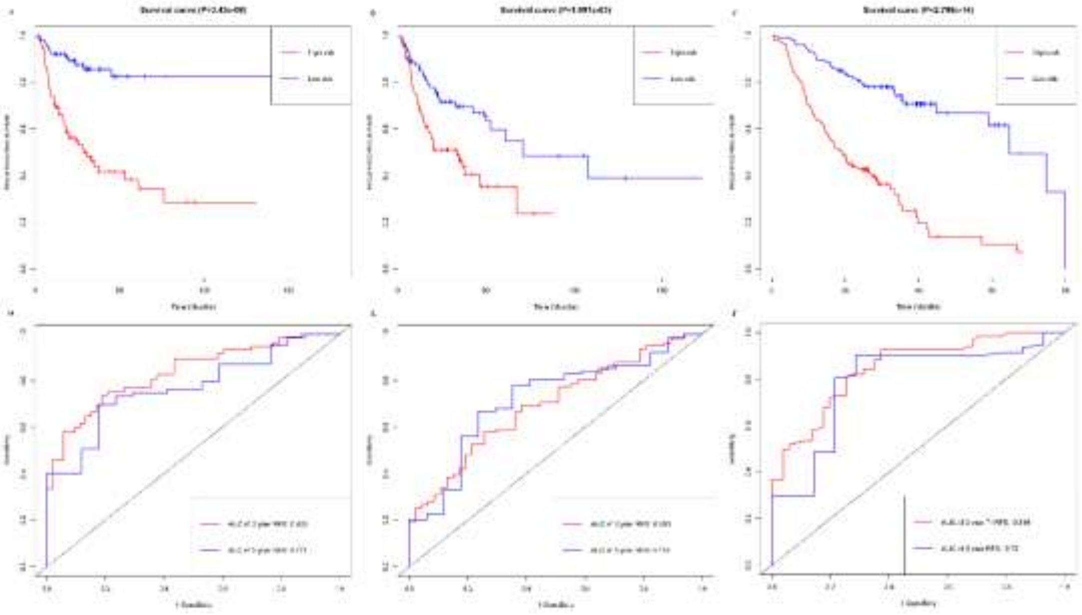

FigureS4: A sensitivity analysis by excluding these cases of oropharynx with time-independent ROC curves to evaluate predictive efficacy of lncRNA classifier risk score (A) in the training set and (B) in the internal validation set.

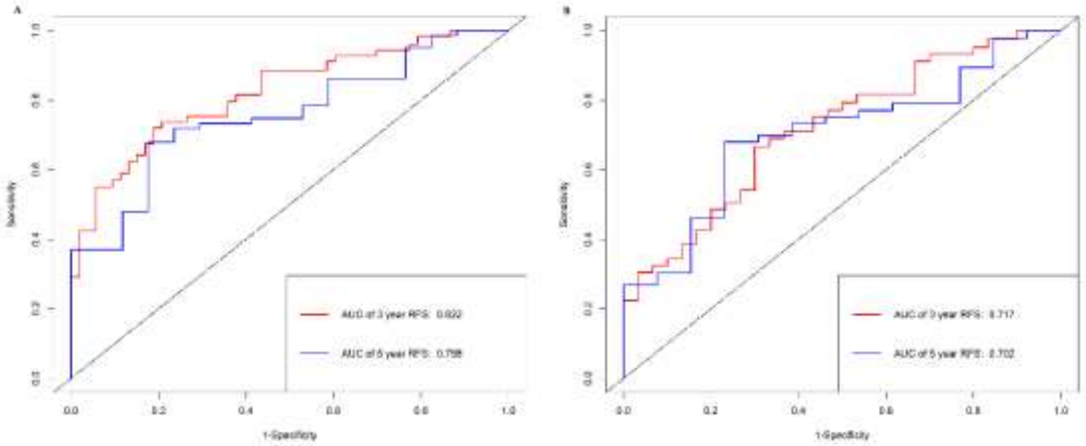

FigureS5. Kaplan–Meier survival analysis according to the lncRNAs signature stratified by clinical factors in the TCGA cohort. (A) HPV status - HPV negative, (B) HPV status - HPV positive; (C) TNM stage- stage I-II, (D) TNM stage- stage III-IV. ***P*** values were calculated using the log-rank test.

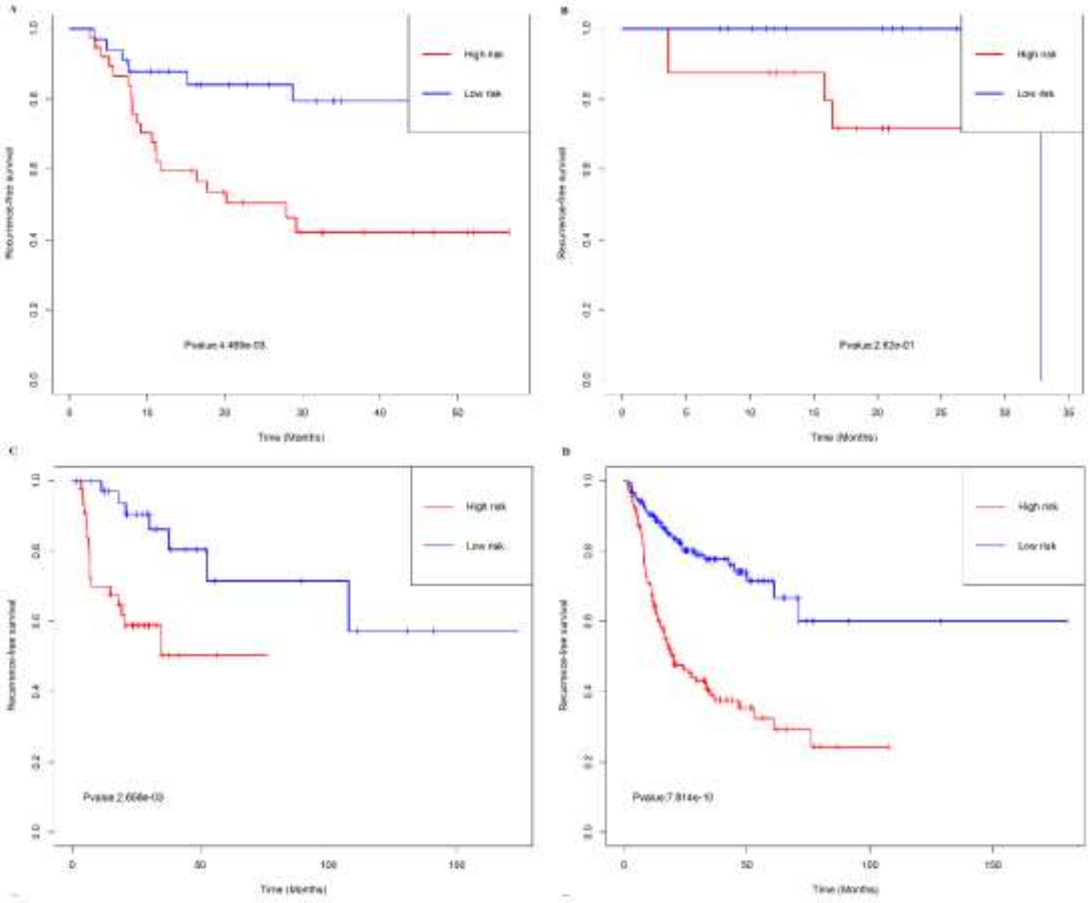

FigureS6 Kaplan–Meier survival analysis according to the lncRNAs signature stratified by clinical factors in the GEO cohort. (A) HPV status - HPV negative, (B) HPV status - HPV positive; (C) TNM stage- stage I-II, (D) TNM stage- stage III-IV. ***P*** values were calculated using the log-rank test.

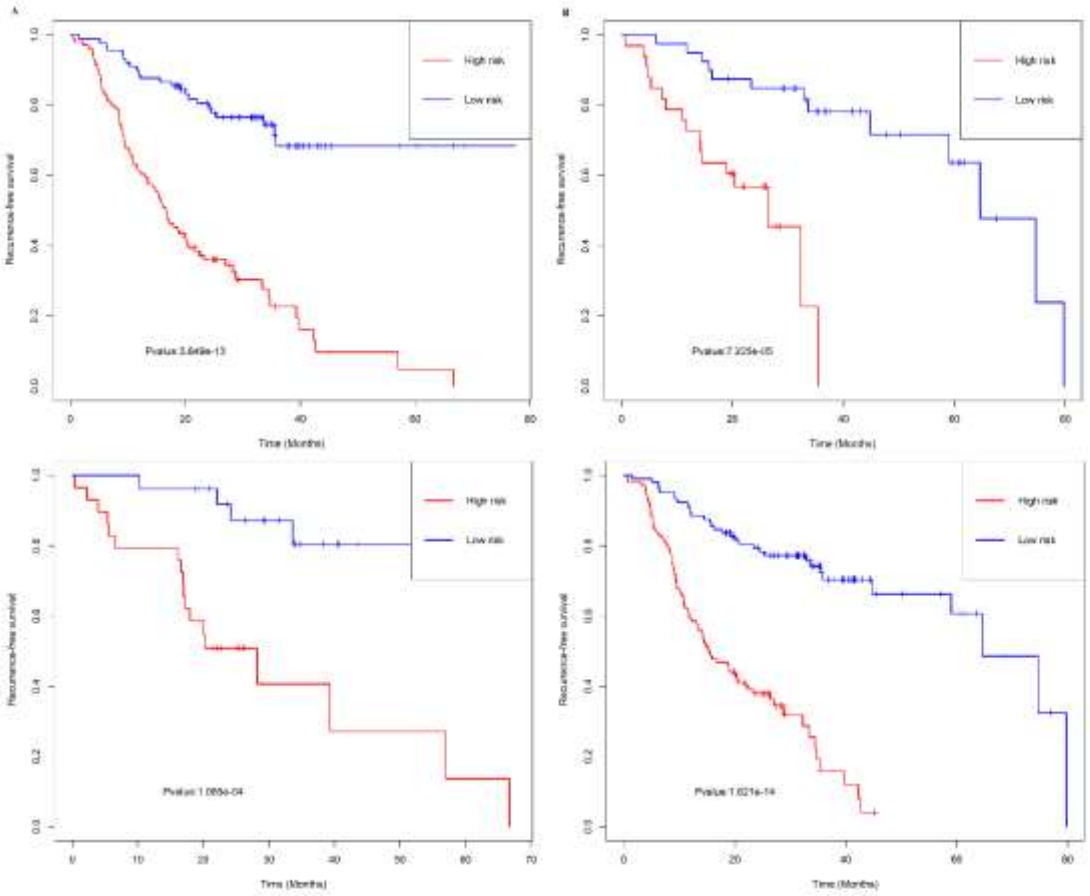

FigureS7. Calibration curves for (A) 3-year nomogram in the training set, and (B) 3-year nomogram in the validation set. Patients were grouped by octiles of predicted risk. x-axis is nomogram-predicted probability of survival(HNSCC). y-axis is observed probability of HNSCC (Kaplan-Meier estimates). Broken line = ideal nomogram; circles = apparent predictive accuracy, calculated by plotting the mean Kaplan-Meier estimate for each octile versus the mean nomogram-predicted probabilities for patients in each octile; X’s = bootstrap-corrected estimates; vertical bars = 95% CIs.

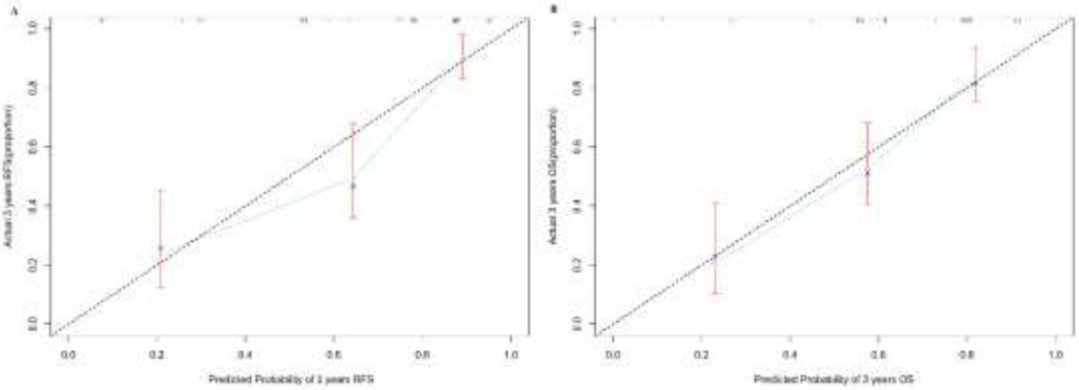

FigureS8: Kaplan-Meier analysis of RFS time for patients of the high-risk subgroup, intermedian risk subgroup and low-risk subgroup stratified by the 5-lncRNAs-based classifier-clinicopathologic nomogram in the training set (A) and in the validation set (B).

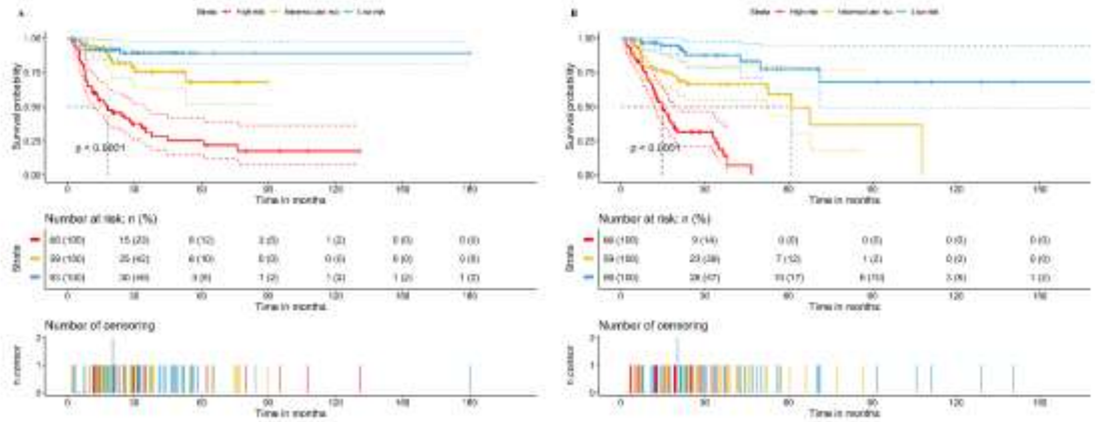

Supplement: Supplementary file 1 — Supplementary Info [file 41598_2019_53811_MOESM1_ESM.pdf]
